# Generalized functional varying-index coefficient model for dynamic synergistic gene-environment interactions with binary longitudinal traits

**DOI:** 10.1371/journal.pone.0318103

**Published:** 2025-01-27

**Authors:** Jingyi Zhang, Honglang Wang, Yuehua Cui

**Affiliations:** 1 Department of Statistics and Probability, Michigan State University, East Lansing, MI, United States of America; 2 Amazon Lab126, Sunnyvale, CA, United States of America; 3 Department of Mathematical Sciences, Indiana University Indianapolis, Indianapolis, IN, United States of America; Semnan University, IRAN, ISLAMIC REPUBLIC OF

## Abstract

The genetic basis of complex traits involves the function of many genes with small effects as well as complex gene-gene and gene-environment interactions. As one of the major players in complex diseases, the role of gene-environment interactions has been increasingly recognized. Motivated by epidemiology studies to evaluate the joint effect of environmental mixtures, we developed a functional varying-index coefficient model (FVICM) to assess the combined effect of environmental mixtures and their interactions with genes, under a longitudinal design with quantitative traits. Built upon the previous work, we extend the FVICM model to accommodate binary longitudinal traits through the development of a generalized functional varying-index coefficient model (gFVICM). This model examines how the genetic effects on a disease trait are nonlinearly influenced by a combination of environmental factors. We derive an estimation procedure for the varying-index coefficient functions using quadratic inference functions combined with penalized splines. A hypothesis testing procedure is proposed to evaluate the significance of the nonparametric index functions. Extensive Monte Carlo simulations are conducted to evaluate the performance of the method under finite samples. The utility of the method is further demonstrated through a case study with a pain sensitivity dataset. SNPs were found to have their effects on blood pressure nonlinearly influenced by a combination of environmental factors.

## 1 Introduction

Longitudinal data analysis is very common in epidemiological studies when the response variables are measured over time on subjects. Numerous studies have demonstrated that longitudinal designs offer greater power in detecting genetic associations compared to cross-sectional designs [1–3]. On the other hand, there has been growing interest in understanding the role of interactions of genes with the environment (G × E) in human diseases, such as type 2 diabetes (e.g., Zimmet et al. [4]) and Parkinson’s disease (e.g., Ross and Smith [5]). In many studies, G × E interactions have traditionally been investigated using a single environment exposure model. Readers are referred to Miao et al. [6] for a comprehensive review of G × E studies. However, increasing evidence suggests that the risk of disease can be significantly influenced by simultaneous exposures to multiple environmental factors. Notably, the combined effect of these exposures often exceeds the sum of their individual effects [7, 8]. This has led to a growing interest in assessing the collective impact of environmental mixtures and exploring the mechanisms through which they interact with genes to influence disease risk. Some research has been done to assess nonlinear interactions between environmental mixtures and genes by applying some nonparametric or semiparametric models, such as the varying index coefficients model (VICM) proposed by [9], the generalized varying index coefficient models by Guo et al. [10], and the partial linear multi-varying index coefficients model (PLMVICM) by Liu et al. [11] and the generalized PLMVICM by Liu et al. [12]. However, these methods were developed for cross-sectional data. The extension of these modeling strategies to longitudinal data deserves further investigation.

Previously, we introduced a functional varying index coefficient model (FVICM) to assess nonlinear G × E interactions for a continuous longitudinal trait [13]. Such trait can be blood pressure or heart rate measured over time. However, in practice, it is possible that the response measured over time is discrete, for example, a binary measure of disease status. In human genetics, many disease traits are binary in nature, as being affected or unaffected (or cases vs controls). To investigate the nonlinear dynamic G × E interaction with environmental mixtures as a whole for a binary longitudinal trait, we propose the following generalized functional varying index coefficient model (gFVICM),

$$\begin{array}{c} g\{E(Y_{ij}|{\text{X}}_{ij},G_{ij})\}=m_{0}\left(\beta_{0}^{T}{\text{X}}_{ij}\right)+m_{1}\left(\beta_{1}^{T}{\text{X}}_{ij}\right)G_{ij}, \end{array}$$ (1)

where *Y*_*ij*_ is the trait of interest observed for the *i*th subject at the *j*th time point (*i* = 1, ⋯, *N*; *j* = 1, ⋯, *n*_*i*_); **X**_*ij*_ is a *p*-dimensional vector of environmental variables, which can be time-variant or time-invariant; *G*_*i*_ denotes a genetic variable which does not depend on time; *g*(⋅) is a known link function which can be the identity link for continuous traits and the logit link for binary traits; *m*_0_(⋅) and *m*_1_(⋅) are unknown nonparametric smooth functions which depend on the data; and ***β***_0_ and ***β***_1_ are *p*-dimensional vectors of index loading parameters. In this work, we consider a binary longitudinal response variable which takes the value of 1 or 0 at each time point. In model (1), the function $m_{1}\left(\beta_{1}^{T}\text{X}\right)$ captures the interaction effect between environmental mixtures and the genetic variable (e.g., a single nucleotide polymorphism (SNP) on the risk of disease).

In this study, we formulate a statistical estimation and hypothesis testing procedure tailored for model (1), specifically for binary longitudinal traits. The Generalized Estimation Equation (GEE) method, proposed by Liang and Zeger [14], has been widely used in longitudinal data analysis. However, there are several disadvantages of GEE method due to some of its critical assumptions [15]. One disadvantage is that the consistency of GEE estimators are based on the consistency of estimators for the nuisance correlation parameter [16, 17]. Another shortcoming of the GEE method is that model selection and hypothesis testing are complicated, because the estimation procedure of the GEE method does not involve an objective function. The quadratic inference function (QIF) approach proposed by Qu et al. [18] is one of the improvements of the GEE method. The QIF method avoids the need to estimate nuisance correlation parameters and has been demonstrated by Qu et al. [18] to be generally more efficient than the GEE method. Furthermore, because the QIF is based on an objective function that is asymptotically chi-square distributed, it naturally accommodates the implementation of model selection criteria such as BIC. The asymptotic property can also allow us to conduct hypothesis tests. This motivates us to extend the QIF method to our model for estimation and hypothesis testing.

Penalized estimation in longitudinal modeling has been extensively studied, to name a few, the penalized GEE [19], penalized GEE with semiparametric generalized mixed-effects partial linear model [20], penalized QIF for varying-coefficient partially linear models [21], and variable selection for nonparametric varying-coefficient models [22]. For methods involving varying-coefficient models, the coefficient functions typically consider only one explanatory variable, which distinguishes them from the one proposed in model (1). In our proposed estimation procedure, we first use penalized splines [23] to approximate the nonparametric smooth functions *m*_*l*_(⋅), *l* = 0, 1. Then, we develop a profile estimation procedure for the index loading parameters and spline coefficients which are estimated iteratively under the QIF framework. In order to avoid overfitting and reduce the number of parameters in spline approximation, we add a penalty to the objective function based on a BIC criterion. We establish the asymptotic normality of the resulting estimators under certain regularity conditions. In addition, we are interested in testing the linearity of G × E interaction, i.e. the linearity of function *m*_1_(⋅). The QIF can be regarded as an inference function which has properties similar to the likelihood ratio test. Based on that, we construct a testing procedure to assess the linearity of the coefficient (interaction) function, where the test statistic asymptotically follows a *χ*^2^ distribution.

The performance of the proposed procedure under finite samples is evaluated by Monte Carlo simulations. The application of the proposed method is demonstrated through the analysis of a pain sensitivity data with a binary response variable indicating whether a subject has hypertension or not (Yes = 1, No = 0). Theories and proofs are rendered in S1 File. Our method offers a novel way for longitudinal G × E study with binary traits in which the focus is on the evaluation of the joint interaction between a genetic variant and a mixture of environmental exposures to affect a disease risk.

## 2 The model and estimation methods

### 2.1 The model

For a binary longitudinal disease trait, suppose the response *y*_*ij*_, the *p*-dimensional covariate vector ***x***_*ij*_, and the SNP variable *G*_*i*_ are observed for the *i*th individual at the *j*th time point, where *i* = 1, ⋯, *N*;*j* = 1, ⋯, *n*_*i*_. In general, the number of potential environmental variables (*p*) that may interact with *G* to affect *Y* is not large. Any other covariates that do no interact with *G* can be modelled separately as a linear term outside of the *m*(⋅) function. Assume that observations from different subjects are independent, but those within the same subject are correlated. We also assume the model satisfies the first moment assumption, i.e.,

$$\begin{array}{c} \mu_{ij}\left(x_{ij},G_{i}\right)=E\left(y_{ij}|x_{ij},G_{i}\right)=g^{-1}\{m_{0}\left(\beta_{0}^{T}x_{ij}\right)+m_{1}\left(\beta_{1}^{T}x_{ij}\right)G_{i}\}, \end{array}$$

where *g*^−1^(⋅) is a given inverse link function. For binary responses, we use a logit link function and the model can be written as

$$\begin{array}{c} \mu_{ij}\left(x_{ij},G_{i}\right)=P\left(y_{ij}=1|x_{ij},G_{i}\right)=\frac{\text{exp}\{m_{0}\left(\beta_{0}^{T}x_{ij}\right)+m_{1}\left(\beta_{1}^{T}x_{ij}\right)G_{i}\}}{1+\text{exp}\{m_{0}\left(\beta_{0}^{T}x_{ij}\right)+m_{1}\left(\beta_{1}^{T}x_{ij}\right)G_{i}\}}. \end{array}$$ (2)

For the identifiability purpose, the constraints ‖***β***_0_‖ = ‖***β***_1_‖ = 1 are imposed, where the first elements of ***β***_0_ and ***β***_1_ are set to be positive.

### 2.2 Quadratic inference function for gFVICM

Denote $u_{0}=\beta_{0}^{T}x$ and $u_{1}=\beta_{1}^{T}x$. First, the unknown coefficient functions *m*_0_(*u*_0_) and *m*_1_(*u*_1_) are approximated by truncated power spline basis as

$$\begin{array}{c} m_{l}\left(u_{l}\right)=m_{l}\left(u_{l},\beta \right)\approx \text{B}{\left(u_{l}\right)}^{T}\gamma_{l},\;\text{for}\;l=0,1, \end{array}$$ (3)

where $\text{B}\left(u\right)={\left(1,u,u^{2},\ldots ,u^{q},{\left(u-\kappa_{1}\right)}_{+}^{q},\ldots ,{\left(u-\kappa_{K}\right)}_{+}^{q}\right)}^{T}$ is a *q*-degree truncated power spline basis with *K* knots *κ*_1_, …, *κ*_*K*_, $\beta ={\left(\beta_{0}^{T},\beta_{1}^{T}\right)}^{T}$, and ***γ***_0_ and ***γ***_1_ are (*q* + *K* + 1)-dimensional vectors of spline coefficients.

A marginal approach such as the GEE, assumes that the marginal mean *μ*_*ij*_ is a function of the covariates through a link function, and the variance of *y*_*ij*_ is a function of the mean var(*y*_*ij*_) = *V*(*μ*_*i*_). The generalized estimation equation for longitudinal data is given as,

$$\begin{array}{c} \sum_{i=1}^{N}{\dot{\mu }}_{i}^{T}{\text{V}}_{i}^{-1}\left({\text{y}}_{i}-\mu_{i}\right)=0, \end{array}$$

where ${\text{y}}_{i}={\left(y_{i1},\ldots ,y_{in_{i}}\right)}^{T}$, ***μ***_*i*_ = *E*(**y**_*i*_) is the mean function, and ${\dot{\mu }}_{i}=\partial \mu_{i}/\partial \theta$ is the first derivative of ***μ***_*i*_ with respect to parameters ***θ*** = (***β***^*T*^, ***γ***^*T*^)^*T*^, with $\gamma ={\left(\gamma_{0}^{T},\gamma_{1}^{T}\right)}^{T}$. The covariance matrix **V**_*i*_ can be decomposed as ${\text{V}}_{i}={\text{A}}_{i}^{1/2}\text{R}\left(\rho \right){\text{A}}_{i}^{1/2}$, where **A**_*i*_ is a diagonal matrix containing the marginal variances, and **R**(*ρ*) is a common working correlation matrix parameterized by a small number of nuisance parameters *ρ*. Utilizing the spline approximation described in Eq (3), the mean function can be expressed as

$$\begin{array}{c} \mu_{i}=\mu_{i}\left(\theta \right)=[\begin{array}{c} \mu_{i1}\left(\theta \right)\\ \vdots \\ \mu_{in_{i}}\left(\theta \right) \end{array}]=[\begin{array}{c} g^{-1}\left\{{\text{B}}^{T}\left(\beta_{0}^{T}x_{i1}\right)\gamma_{0}+{\text{B}}^{T}\left(\beta_{1}^{T}x_{i1}\right)\gamma_{1}G_{i}\right\}\\ \vdots \\ g^{-1}\left\{{\text{B}}^{T}\left(\beta_{0}^{T}x_{in_{i}}\right)\gamma_{0}+{\text{B}}^{T}\left(\beta_{1}^{T}x_{in_{i}}\right)\gamma_{1}G_{i}\right\} \end{array}], \end{array}$$

and the first derivative of ***μ***_*i*_ is

$$\begin{array}{c} {\dot{\mu }}_{i}=[\begin{array}{cccc} {\left(g^{-1}\right)}^{\prime }{\text{B}}_{d}^{T}\left(\beta_{0}^{T}x_{i1}\right)\gamma_{0}x_{i1}^{T} & {\left(g^{-1}\right)}^{\prime }{\text{B}}_{d}^{T}\left(\beta_{1}^{T}x_{i1}\right)\gamma_{1}G_{i}x_{i1}^{T} & {\left(g^{-1}\right)}^{\prime }{\text{B}}^{T}\left(\beta_{0}^{T}x_{i1}\right) & {\left(g^{-1}\right)}^{\prime }{\text{B}}^{T}\left(\beta_{1}^{T}x_{i1}\right)G_{i}\\ \vdots & \vdots & \vdots & \vdots \\ {\left(g^{-1}\right)}^{\prime }{\text{B}}_{d}^{T}\left(\beta_{0}^{T}x_{in_{i}}\right)\gamma_{0}x_{in_{i}}^{T} & {\left(g^{-1}\right)}^{\prime }{\text{B}}_{d}^{T}\left(\beta_{1}^{T}x_{in_{i}}\right)\gamma_{1}G_{i}x_{in_{i}}^{T} & {\left(g^{-1}\right)}^{\prime }{\text{B}}^{T}\left(\beta_{0}^{T}x_{in_{i}}\right) & {\left(g^{-1}\right)}^{\prime }{\text{B}}^{T}\left(\beta_{1}^{T}x_{in_{i}}\right)G_{i} \end{array}], \end{array}$$

where ${\text{B}}_{d}\left(u\right)=\partial \text{B}\left(u\right)/\partial u=\left(0,1,2u,\ldots ,qu^{q-1},q{\left(u-\kappa_{1}\right)}_{+}^{q-1},\ldots ,q{\left(u-\kappa_{K}\right)}_{+}^{q-1}\right)$.

In the QIF method, the inverse of the working correlation matrix can be approximated by a linear combination of several basis matrices [18]. This can be represented as:

$${\text{R}}^{-1}\left(\rho \right)\approx a_{1}{\text{M}}_{1}+\ldots +a_{h}{\text{M}}_{h},$$

where **M**_1_ is the identity matrix, and **M**_2_, …, **M**_*h*_ are predefined basis matrices. For instance:

- If the working correlation is exchangeable, **R**^−1^ ≈ *a*_1_**M**_1_ + *a*_2_**M**_2_, where **M**_2_ has zeros on the diagonal and ones on the off-diagonal.
- If the working correlation follows an AR(1) structure, ${\text{R}}^{-1}\approx a_{1}^{*}{\text{M}}_{1}+a_{2}^{*}{\text{M}}_{2}^{*}$ with ${\text{M}}_{2}^{*}$ having ones on its two subdiagonals and zeros elsewhere.

The advantage of this approach is that it simplifies the estimation process by eliminating the need to directly estimate the nuisance parameters *a*_1_, …, *a*_*h*_.

Building on this concept, we can formulate the estimation function as follows:

$$\begin{array}{c} {\bar{g}}_{N}\left(\theta \right)=\frac{1}{N}\sum_{i=1}^{N}g_{i}\left(\theta \right)=\frac{1}{N}[\begin{array}{c} \sum_{i=1}^{N}{\dot{\mu }}_{i}^{T}{\text{A}}_{i}^{-1/2}{\text{M}}_{1}{\text{A}}_{i}^{-1/2}\left({\text{y}}_{i}-\mu_{i}\right)\\ \vdots \\ \sum_{i=1}^{N}{\dot{\mu }}_{i}^{T}{\text{A}}_{i}^{-1/2}{\text{M}}_{h}{\text{A}}_{i}^{-1/2}\left({\text{y}}_{i}-\mu_{i}\right) \end{array}]. \end{array}$$ (4)

Since the number of equations in ${\bar{g}}_{N}$ (2*ph* + 2(*q* + *K* + 1)*h*) exceeds the number of unknown parameters (2*p* + 2(*q* + *K* + 1)), we cannot solve for the estimators by merely setting each element to zero. To address this, we estimate the parameters by minimizing the following quadratic inference function:

$$\begin{array}{c} Q_{N}\left(\theta \right)=N{\bar{g}}_{N}^{T}{\bar{C}}_{N}^{-1}{\bar{g}}_{N}, \end{array}$$

where ${\bar{C}}_{N}=\frac{1}{N}\sum_{i=1}^{N}g_{i}g_{i}^{T}$ serves as a consistent estimator for var(*g*_*i*_). By minimizing the quadratic inference function, we can derive the parameter estimates as follows:

$$\begin{array}{c} \hat{\theta }=\text{arg}\;\underset{\theta }{\text{min}}\;Q_{N}\left(\theta \right). \end{array}$$

In order to avoid over-parameterization, we add a penalty term to QIF to penalize the number of knots [24]. The penalized QIF is written as

$$\begin{array}{c} N^{-1}Q_{N}\left(\theta \right)+\lambda \theta^{T}\text{D}\theta , \end{array}$$ (5)

where **D** is a diagonal matrix with 1 for parameters corresponding to spline coefficients associated with knots and 0 otherwise. Specifically, $\text{D}=\text{diag}(0_{(2p+q+1)\times 1}^{T},1_{K\times 1}^{T},0_{(q+1)\times 1}^{T},1_{K\times 1}^{T})$. Thus, the estimator is given by:

$$\begin{array}{c} \hat{\theta }=\text{arg}\;\underset{\theta }{\text{min}}\{N^{-1}Q_{N}\left(\theta \right)+\lambda \theta^{T}\text{D}\theta \}. \end{array}$$ (6)

To determine the tuning parameter λ, we borrow the generalized cross-validation idea [24–26]. The generalized cross-validation statistic is defined as

$$\begin{array}{c} \text{GCV}\left(\lambda \right)=\frac{N^{-1}Q_{N}}{{\left(1-N^{-1}\text{df}\right)}^{2}} \end{array}$$

where the effective degree of freedom is given by $\text{df}=\text{tr}\{{\left({\ddot{Q}}_{N}+2N\lambda D\right)}^{-1}{\ddot{Q}}_{N}\}$, ${\ddot{Q}}_{N}$ represents the second derivative of *Q*_*N*_. The optimal tuning parameter λ is the one that minimizes GCV(λ). In the process of implementing GCV, the desired value of λ can be found using a grid search by predefining a set of values for λ. We also established the asymptotic properties for the estimators of the index loading parameters and the penalized spline regression coefficients which are given in the online S1 File together with the proof.

## 3 Model selection and hypothesis test

### 3.1 Model selection

Model selection is crucial in spline approximation, as including too many parameters can lead to overfitting. According to the theoretical property of the generalized method of moments estimator [27], under the assumption that *E*(*g*_1_) = 0 and the number of estimating equations exceeds the number of parameters, we have $Q\left(\hat{\theta }\right)\to \chi_{r-k}^{2}$ in distribution. Here, *r* is the dimension of ${\bar{g}}_{N}\left(\theta \right)$, *k* is the dimension of ***θ***, and $\hat{\theta }$ is the estimator obtained by minimizing the QIF given a specific order and number of knots. This asymptotic property of the QIF allows for a goodness-of-fit test, which is useful in determining the appropriate order and number of knots for our model. However, multiple models that are not nested may pass the goodness-of-fit tests. Given that $Q(\hat{\theta })$ is asymptotically chi-square distributed, it is natural to extend the BIC to the QIF approach, by replacing twice the negative log-likelihood function by the QIF objective function [28]. Specifically, the BIC criterion for a model with *r* estimating equations and *k* parameters is expressed as follows:

$$\begin{array}{c} Q\left(\hat{\theta }\right)+\left(r-k\right)\text{ln}\;N. \end{array}$$

The model with the lowest BIC is deemed be the optimal choice. If we select *h* basis matrices in (4), then *r* − *k* = *hk* − *k* = (*h* − 1)*k*.

In our simulation and real data application, we determine the number of knots *K* and the optimal order *q* by exploring various combinations and selecting the one that minimizes the BIC criterion. The knots are evenly spaced across the range of the single index *u* = ***β***^*T*^**X**.

### 3.2 Nonparametric goodness-of-fit test based on QIF

The QIF can also be regarded as an inference function since it has properties similar to the likelihood ratio test. Suppose that the *d*-dimensional parameter vector ***γ*** is partitioned into (***ψ***, ***ζ***), where ***ψ*** is the parameter of interest with dimension *d*_1_, and ***ζ*** is the nuisance parameter with dimension *d*_2_ = *d* − *d*_1_. If we are interested in testing

$$\begin{array}{c} H_{0}:\psi =\psi_{0}, \end{array}$$

the test statistic

$$\begin{array}{c} Q\left(\psi_{0},\tilde{\zeta }\right)-Q\left(\hat{\psi },\hat{\zeta }\right) \end{array}$$

follows an asymptotically chi-square distribution with *d*_1_ degrees of freedom. Qu et al. [18] introduced a theorem that provided a way to conduct hypothesis testing in the QIF framework. The theorem states that given that all required regularity conditions are satisfied and ***ψ*** has dimension *d*_1_, under the null hypothesis, $Q\left(\psi_{0},\tilde{\zeta }\right)-Q\left(\hat{\psi },\hat{\zeta }\right)$ is asymptotically chi-square distribution with *d*_1_ degrees of freedom, where

$$\begin{array}{c} \tilde{\zeta }=\text{arg}\;\text{min}\;Q\left(\psi_{0},\zeta \right),\;\;\;\left(\hat{\psi },\hat{\zeta }\right)=\text{arg}\;\text{min}\;Q\left(\psi ,\zeta \right). \end{array}$$ (7)

When there is no nuisance parameter, which is a special case of the condition in the theorem, $Q\left(\gamma_{0}\right)-Q\left(\hat{\gamma }\right)$ has an asymptotical chi-square distribution with *d* degree of freedom under the null hypothesis.

### 3.3 Test for linearity of the interaction function in gFVICM

In our proposed gFVICM model as outlined in Eq (1), a key focus is to test the form of the unspecified coefficient function. Specifically, we are interested in determining whether a linear function is sufficient to describe the G × E interaction. If we fail to reject the hypothesis that the coefficient function is linear, we should fit a parametric linear interaction model to further evaluate the presence of a linear G × E interaction. Conversely, if the hypothesis is rejected, it suggests the presence of a nonlinear G × E interaction. It is important to note that we cannot directly test for the zero effect of the function *m*_1_(⋅) because, under the null hypothesis *m*_1_(⋅) = 0, the index loading parameters become unidentifiable unless we impose the condition ***β***_0_ = ***β***_1_ = ***β*** which is practically too restrictive. Let $u_{1}=\beta_{1}^{T}\text{X}$. Using the truncated power spline basis, the coefficient function can be approximated as:

$$\begin{array}{c} m_{1}\left(u_{1}\right)\approx \gamma_{10}+\gamma_{11}u_{1}+\gamma_{12}u_{1}^{2}+\cdots +\gamma_{1q}u_{1}^{q}+\sum_{k=q+1}^{K+q+1}\gamma_{1k}{\left(u_{1}-\kappa_{k}\right)}_{+}^{q}. \end{array}$$

Our objective is to test the linearity of *m*_1_(*u*_1_), which is equivalent to testing

$$\begin{array}{c} H_{0}:\gamma_{12}=\cdots =\gamma_{1,K+q+1}=0. \end{array}$$

Let $\tilde{\theta }$ be the estimator of the full parameter ***θ*** = (***β***^*T*^, ***γ***^*T*^)^*T*^ under the null hypothesis with

$$\begin{array}{c} \tilde{\theta }={\left({\tilde{\beta }}^{T},{\tilde{\gamma }}_{0}^{T},{\tilde{\gamma }}_{10},{\tilde{\gamma }}_{11},0^{T}\right)}^{T}=\underset{\gamma_{12}=\cdots =\gamma_{1,K+q+1}=0}{\text{arg}\;\text{min}}Q_{N}\left(\theta \right), \end{array}$$

and the estimator of ***θ*** under the alternative as $\hat{\theta }=\text{arg}\;\text{min}\;Q_{N}\left(\theta \right)$. Then following the theorem by Qu et al. [18], the test statistic

$$\begin{array}{c} T_{N}=Q_{N}\left(\tilde{\theta }\right)-Q_{N}\left(\hat{\theta }\right), \end{array}$$

asymptotically follows a chi-square distribution with *K* + *q* − 1 degrees of freedom.

## 4 Simulation study

The performance of the proposed method in finite samples was assessed through Monte Carlo simulation studies. Specifically, we examined the following logistic regression model:

$$\begin{array}{c} P\left(y_{ij}=1|{\text{X}}_{ij},G_{i},\beta \right)=\frac{\text{exp}\{\eta \left({\text{X}}_{ij},G_{i},\beta \right)\}}{1+\text{exp}\{\eta \left({\text{X}}_{ij},G_{i},\beta \right)\}}, \end{array}$$

where

$$\begin{array}{c} \eta \left({\text{X}}_{ij},G_{i},\beta \right)=m_{0}\left(\beta_{0}^{T}{\text{X}}_{ij}\right)+m_{1}\left(\beta_{1}^{T}{\text{X}}_{ij}\right)G_{i}. \end{array}$$

We simulated a three-dimensional set of environmental variables **X** = (*X*_1_, *X*_2_, *X*_3_). For each subject *i*, the variables *X*_1*ij*_, *X*_2*ij*_, *X*_3*ij*_ were independently drawn from a uniform distribution *U*(0, 1). We set the minor allele frequency (MAF) to *p*_*A*_ = 0.1, 0.3, 0.5, assuming Hardy-Weinberg equilibrium. The SNP genotypes *AA*, *Aa*, and *aa* were simulated based on a multinomial distribution with probabilities $p_{A}^{2}$, 2*p*_*A*_(1 − *p*_*A*_) and (1 − *p*_*A*_)^2^, respectively. The genotype variable *G* was encoded as {0,1,2}, corresponding to the genotypes {*aa*, *Aa*, *AA*}, respectively. To generate correlated responses, we implemented the R package bindata developed by Leisch et al. [29] under an AR(1) correlation structure with correlation parameter *ρ* = 0.5. When implementing the function ‘rmvbin’ to generate the correlated binary data, one should specify the marginal probabilities and the correlation structure.

We set *m*_0_(*u*_0_) = cos(*πu*_0_) and *m*_1_(*u*_1_) = sin[*π*(*u*_1_ − *A*)/(*B* − *A*)] with $A=\sqrt{3}/2-1.645/\sqrt{12}$ and $B=\sqrt{3}/2+1.645/\sqrt{12}$. The true parameters were $\beta_{0}=\left(\sqrt{5},\sqrt{4},\sqrt{4}\right)/\sqrt{13}$ and $\beta_{1}=\left(1,1,1\right)/\sqrt{3}$. We generated 500 data sets, each with a sample size *N* = 200 or 500, and observed at time points *n*_*i*_ = *T* = 10 or 20, respectively. For simplicity, we assumed all subjects were measured at equal amount of time points though this assumption is not required. The basis matrix **M**_2_ was set to have 1 on its two subdiagonals and 0 elsewhere. The number and order of knots for the splines were determined based on the BIC criterion.

### 4.1 Performance of estimation

Tables 1 and 2 present the parameter estimation results for different sample sizes and measurement times, respectively. These tables report the average bias (Bias), the average of the estimated standard error (SE) derived from the theoretical results, the standard deviation of the 500 estimates (SD), and the estimated coverage probability (CP) at the 95% confidence level. The tables show that as the sample size increases, the performance of the estimation improves, evidenced by reduced bias, SD, and SE. Additionally, increasing the number of repeated measurements for each subject also enhances estimation accuracy, as illustrated by the comparison between Tables 1 and 2. For instance, the CP for *β*_01_ improves from 86.8% to 90% when the number of measurement times increases from 10 to 20, given a sample size of 200. Moreover, the estimation of the loading parameter ***β***_1_ becomes more accurate as MAF *p*_*A*_ increases. Conversely, the estimation of ***β***_0_ tends to deteriorate with high *p*_*A*_. This is likely because we have less data information for accurately estimating the marginal effects *m*_0_(⋅) when *p*_*A*_ is large.

**Table 1:** Simulation results under different MAFs (*p*_*A*_ = 0.1, 0.3, 0.5) and sample sizes (*N* = 200, 500), *T* = 10 and correlation *ρ* = 0.5.

*N*	Param	True	*p*_*A*_ = 0.1				*p*_*A*_ = 0.3				*p*_*A*_ = 0.5			
			Bias	SD	SE	CP	Bias	SD	SE	CP	Bias	SD	SE	CP
200	*β*_01_	0.620	-0.008	0.058	0.057	93.6	-0.003	0.074	0.064	90.6	-0.007	0.084	0.068	86.8
	*β*_02_	0.555	-0.002	0.066	0.056	90.0	-0.004	0.080	0.062	87.2	-0.008	0.093	0.065	84.5
	*β*_03_	0.555	-4.1E-05	0.064	0.056	91.8	-0.008	0.074	0.062	88.8	-0.006	0.094	0.066	86.6
	*β*_11_	0.577	-0.013	0.134	0.091	82.2	-0.003	0.092	0.072	87.2	0.007	0.088	0.063	84.9
	*β*_12_	0.577	-0.024	0.139	0.090	79.2	-0.10	0.096	0.071	85.2	-0.013	0.085	0.062	87.2
	*β*_13_	0.577	-0.013	0.140	0.091	82.4	-0.11	0.095	0.071	86.0	-0.014	0.088	0.062	85.4
500	*β*_01_	0.620	0.002	0.038	0.038	94.8	-0.002	0.043	0.043	95.4	-0.003	0.047	0.048	95.0
	*β*_02_	0.555	0.003	0.039	0.037	93.0	-0.002	0.043	0.042	93.4	-2.1E-04	0.052	0.046	92.4
	*β*_03_	0.555	-0.003	0.038	0.037	93.8	-6.3E-04	0.045	0.042	93.0	-0.004	0.050	0.046	92.5
	*β*_11_	0.577	-0.007	0.078	0.065	89.6	-0.002	0.055	0.049	92.0	0.002	0.045	0.044	94.2
	*β*_12_	0.577	-0.003	0.079	0.066	88.0	-0.001	0.052	0.049	92.8	-0.003	0.049	0.043	91.4
	*β*_13_	0.577	-0.005	0.075	0.066	90.6	-0.004	0.054	0.049	92.8	-0.005	0.047	0.043	92.0

**Table 2:** Simulation results under different MAFs (*p*_*A*_ = 0.1, 0.3, 0.5) and sample sizes (*N* = 200, 500), *T* = 20 and correlation *ρ* = 0.5.

*N*	Param	True	*p*_*A*_ = 0.1				*p*_*A*_ = 0.3				*p*_*A*_ = 0.5			
			Bias	SD	SE	CP	Bias	SD	SE	CP	Bias	SD	SE	CP
200	*β*_01_	0.620	-0.002	0.044	0.043	94.4	-0.002	0.053	0.049	94.2	-0.011	0.062	0.052	90.0
	*β*_02_	0.555	-0.004	0.048	0.042	90.2	-0.003	0.056	0.048	90.2	-0.003	0.065	0.050	88.3
	*β*_03_	0.555	2.3E-04	0.048	0.042	91.2	-0.002	0.057	0.048	90.2	0.005	0.063	0.050	87.4
	*β*_11_	0.577	-0.006	0.074	0.064	90.0	0.008	0.062	0.054	91.8	0.004	0.063	0.050	88.2
	*β*_12_	0.577	-0.004	0.075	0.064	91.4	-0.009	0.063	0.053	89.8	-0.009	0.064	0.050	87.1
	*β*_13_	0.577	-0.005	0.072	0.064	91.8	-0.009	0.063	0.054	90.0	-0.006	0.064	0.050	86.8
500	*β*_01_	0.620	-0.002	0.028	0.028	97.2	-0.002	0.033	0.033	95.0	-0.004	0.037	0.036	93.2
	*β*_02_	0.555	-3.2E04	0.028	0.027	94.8	0.001	0.033	0.032	94.0	7.9E-05	0.038	0.035	92.8
	*β*_03_	0.555	3.5E04	0.029	0.027	93.2	-0.002	0.034	0.032	92.8	6.3E-04	0.037	0.035	94.2
	*β*_11_	0.577	-0.005	0.044	0.044	92.2	0.004	0.039	0.036	92.4	0.006	0.038	0.035	93.0
	*β*_12_	0.577	-0.002	0.045	0.045	91.0	-0.003	0.037	0.036	93.0	-0.004	0.036	0.035	94.2
	*β*_13_	0.577	0.003	0.042	0.042	96.4	-0.005	0.038	0.036	93.4	-0.005	0.035	0.035	93.6

**Fig 1:**
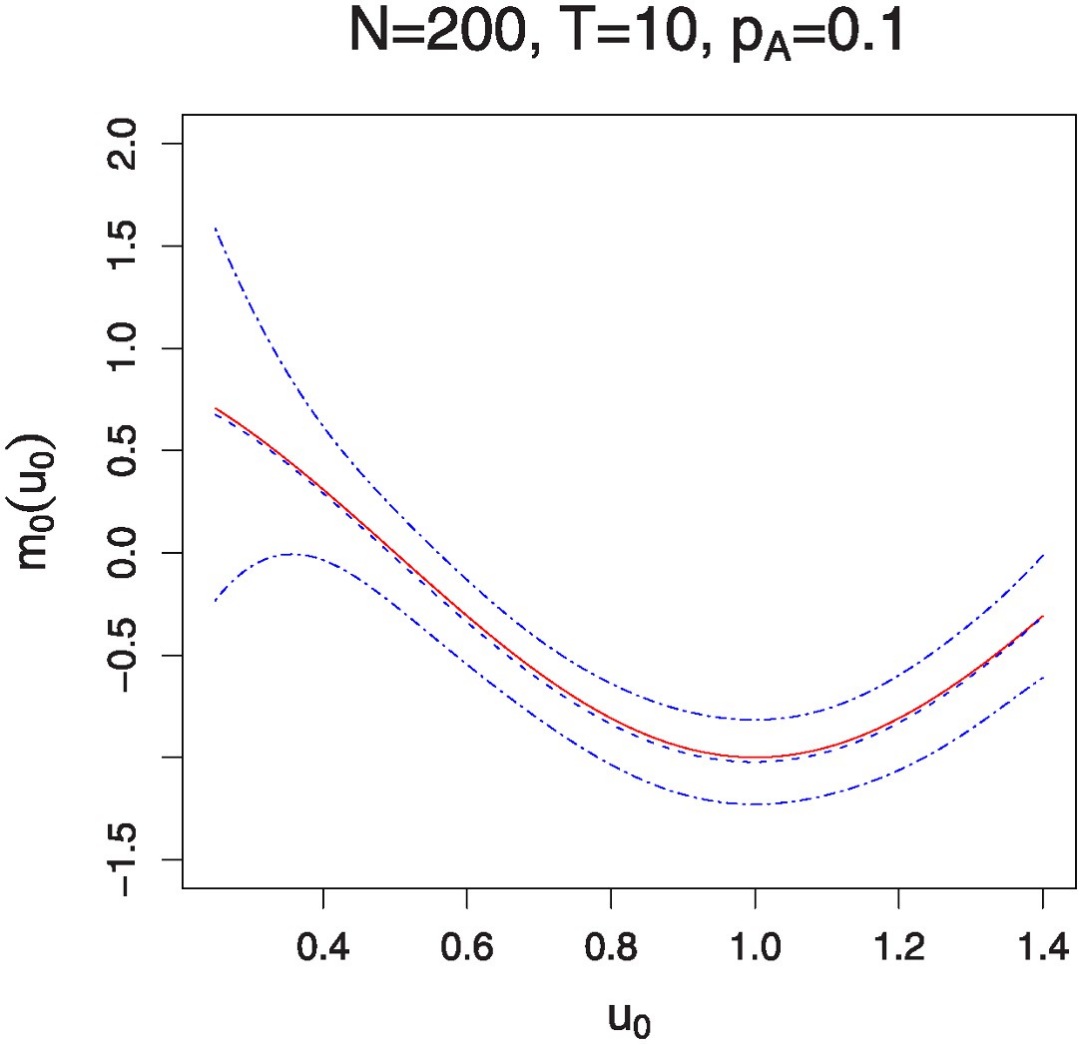
The estimation of function *m*_0_(⋅) for sample sizes *N* = 200 and 500 with *T* = 10 time points. The solid lines represent the estimated functions, while the dashed lines denote the true functions. The 95% confidence bands are illustrated by the dotted-dash lines.

**Fig 2:**
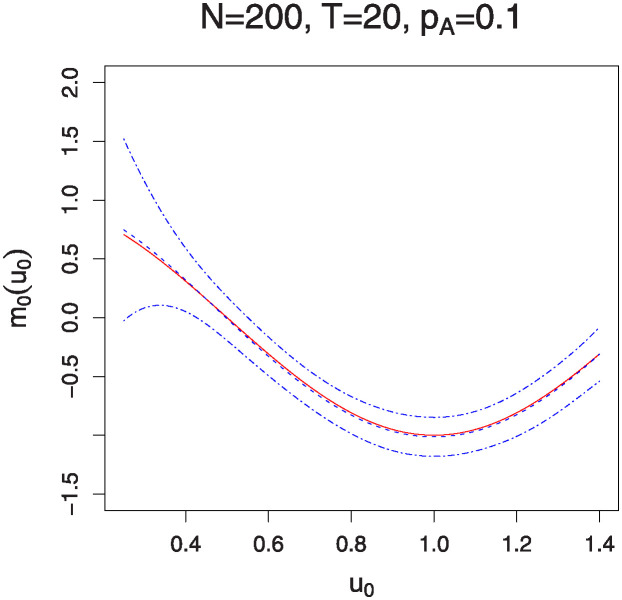
The estimation of function *m*_0_(⋅) for sample sizes *N* = 200 and 500 with *T* = 20 time points. The solid lines represent the estimated functions, while the dashed lines denote the true functions. The 95% confidence bands are illustrated by the dotted-dash lines.

**Fig 3:**
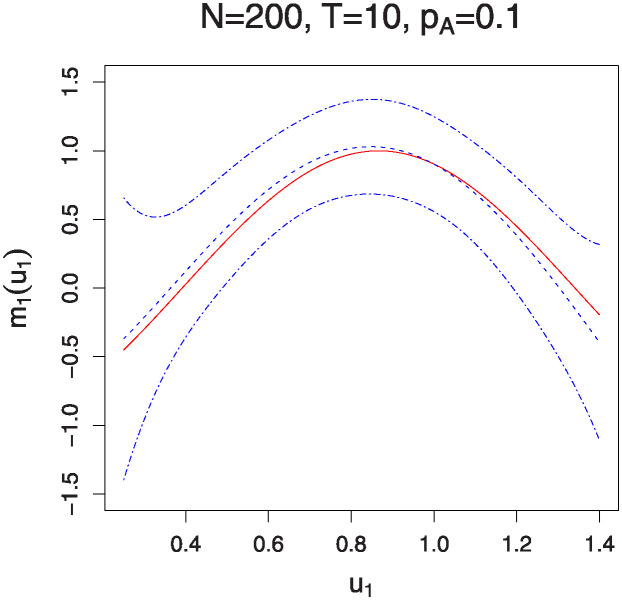
The estimation of function *m*_1_(⋅) for sample sizes *N* = 200 and 500 with *T* = 10 time points. The solid lines represent the estimated functions, while the dashed lines denote the true functions. The 95% confidence bands are illustrated by the dotted-dash lines.

**Fig 4:**
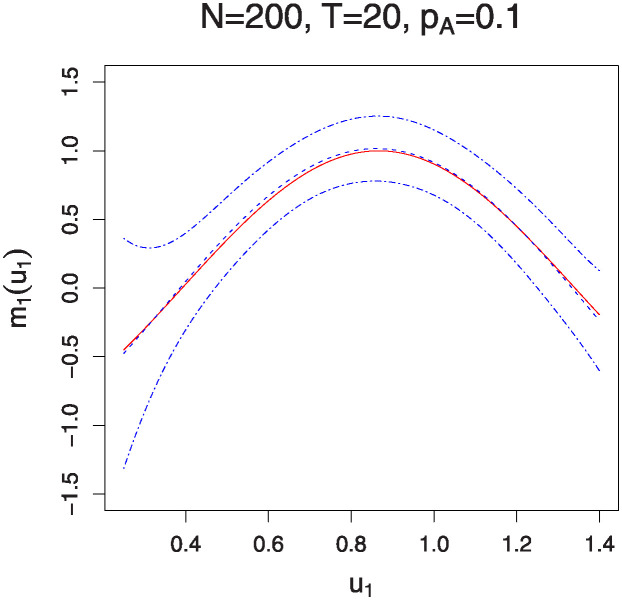
The estimation of function *m*_1_(⋅) for sample sizes *N* = 200 and 500 with *T* = 20 time points. The solid lines represent the estimated functions, while the dashed lines denote the true functions. The 95% confidence bands are illustrated by the dotted-dash lines.

Figs 1–4 illustrate the estimated functions *m*_0_(*u*_0_) and *m*_1_(*u*_1_) under varying sample sizes and time points. In these plots, the solid lines represent the estimated functions, while the dashed lines denote the true functions. The 95% confidence bands are indicated by the dotted-dash lines. The plots show that the estimated curves almost perfectly align with the true curves, demonstrating the high accuracy of the estimation method. Additionally, the confidence bands are particularly tight for larger sample sizes and a greater number of measurement times, indicating robust estimation. It is noteworthy that the estimation of the interaction effects *m*_1_(*u*_1_) improves with an increase in *p*_*A*_. Conversely, the estimation of the marginal effects *m*_0_(*u*_0_) becomes less accurate as *p*_*A*_ increases. This observation aligns with the parameter estimation results presented in Tables 1 and 2.

### 4.2 Performance of hypothesis tests

We assessed the performance of the test for the nonparametric function under the null hypothesis $H_{0}:m_{1}\left(\cdot \right)=m_{1}^{0}\left(\cdot \right)$, where $m_{1}^{0}\left(u_{1}\right)=\delta_{0}+\delta_{1}u_{1}$, *δ*_0_ and *δ*_1_ are constants, representing a linear G × E interaction. To evaluate the test’s power, we considered a sequence of alternative models denoted by $H_{1}^{\tau }:m_{1}^{\tau }\left(\cdot \right)=m_{1}^{0}\left(\cdot \right)+\tau \left\{m_{1}\left(\cdot \right)-m_{1}^{0}\left(\cdot \right)\right\}$, where *τ* varies. When *τ* = 0, the test evaluates the false positive rate.

**Fig 5:**
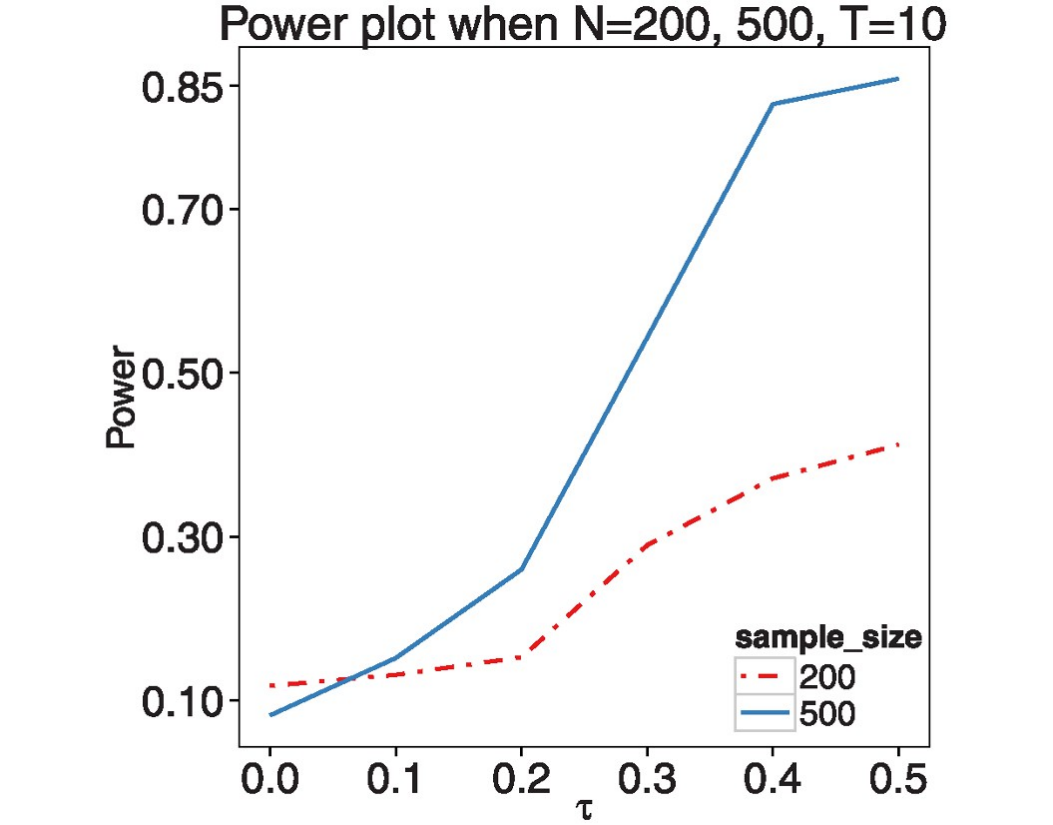
The empirical size and power of testing the linearity of nonparametric function *m*_1_(⋅) for sample sizes *N* = 200 and 500 with *T* = 10 (left) and 20(right) time points.

Fig 5 illustrates the empirical size (for *τ* = 0) and power (for *τ* > 0) of the test at the 5% significance level, based on 500 Monte Carlo simulations. The analysis was conducted for sample sizes *N* = 200 and 500, under different measurement times: *T* = 10 (left panel) and *T* = 20 (right panel), with MAF fixed at 0.3. When the sample size is *N* = 200, the empirical Type I error rate is relatively high. However, this rate decreases significantly as the sample size increases to *N* = 500. Additionally, the power of the test improves markedly with an increase in sample size from 200 to 500. These findings suggest that our method effectively controls false positive rates and exhibits adequate power to detect deviations from the linear function, particularly under larger sample sizes. Moreover, a comparison between the results for *T* = 10 and *T* = 20 shows that the testing power increases with more frequent measurements, indicating better performance in detecting nonlinearity with higher measurement frequency.

**Fig 6:**
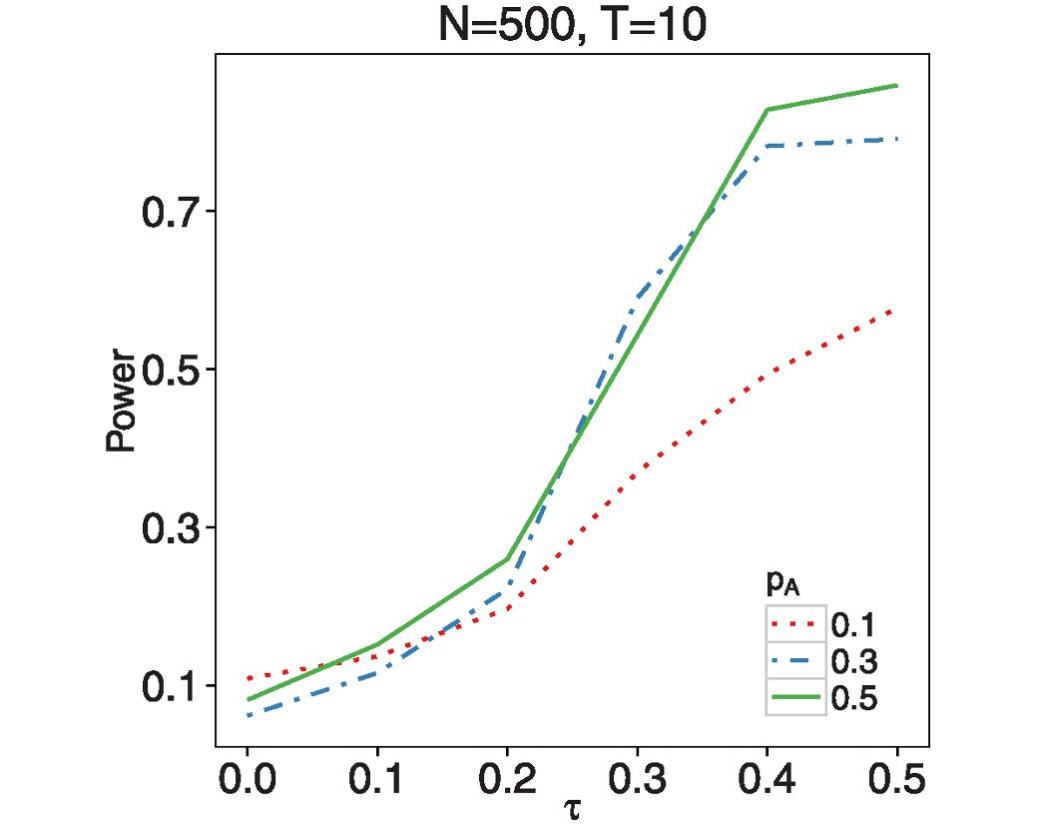
The empirical size and power of testing the linearity of nonparametric function *m*_1_ under different MAFs for *N* = 500 and *T* = 10.

To assess how the values of MAF affect the testing performance, we plotted the power under different MAFs *p*_*A*_ = 0.1, 0.3, 0.5 when *N* = 500, *T* = 10, which is shown in Fig 6. The power of the test increases significantly when MAF rises from 0.1 to 0.3. However, the power values are quite similar when *p*_*A*_ = 0.3 and 0.5.

## 5 Real data application

We applied the proposed gFVICM model to a dataset from a study investigating the association between the A118G SNPs of the OPRM1 gene and sensitivity to experimental pain. A sample of 163 healthy volunteers were recruited to this study. For each volunteer, Systolic Blood Pressure (SBP) and Diastolic Blood Pressure (DBP) were measured at 6 Dobutamine dosage levels: 0 (baseline), 5, 10, 20, 30 and 40mcg/min. Missing values were present in genotypes, covariates, and disease responses, with all missing rates falling below 10%. Rather than excluding the observations, given the small sample size of the study, we opted to impute the missing values before conducting the analysis. Clinically, a person is said to be hypertensive if the individual’s SBP is greater than 140mm Hg or DBP is greater than 90mm Hg [30]. Thus, the response variable *Y* is a binary variable indicating whether a person has hypertension or not, i.e. *Y* = 1 for hypertension and *Y* = 0 for non-hypertension. The model with the mean given in (2) is applied to the data.

One longitudinal covariate *X*_1_ = dosage level, two time-invariant covariates *X*_2_ = age and *X*_3_ = BMI were included as the environmental factors in the model. The genetic variables were five SNPs located at codon 16, 27, 49, 389, and 492 in the gene. Our goal was to assess how a combination of age, BMI and dosage level modifies the effect of the SNP on the risk of hypertension. Specifically, we tested the hypothesis *H*_0_: *m*_1_(*u*_1_) = *δ*_0_ + *δ*_1_*u*_1_ with the corresponding p-value denoted as $p_{m_{1}}$. P-values for testing the significance of the three index loading coefficients ***β***_1_ = (*β*_11_, *β*_12_, *β*_13_) were also reported and labeled as $p_{\beta_{11}}$, $p_{\beta_{12}}$, and $p_{\beta_{13}}$, respectively, following the asymptotic property of the estimators. Additionally, our proposed model was compared with a generalized additive varying-coefficient model (gAVCM) formulated as $E\left(Y|\text{X},G\right)=\eta \{\beta_{01}^{*}\left(X_{1}\right)+\beta_{02}^{*}X_{2}+\beta_{03}^{*}X_{3}+(\beta_{11}^{*}\left(X_{1}\right)+\beta_{12}^{*}X_{2}+\beta_{13}^{*}X_{3})G\}$, where $\beta_{01}^{*}\left(\cdot \right)$ and $\beta_{11}^{*}\left(\cdot \right)$ are unknown functions of *X*_1_. To evaluate the relative benefits of our integrative analysis, we calculated the objective function *Q*_*N*_ for both models. The p-values for testing $H_{0}:\beta_{11}^{*}\left(\cdot \right)=\beta_{12}^{*}=\beta_{13}^{*}=0$ in the gAVCM are also provided in the tables and are denoted by *p*_*gAVCM*_.

**Fig 7:**
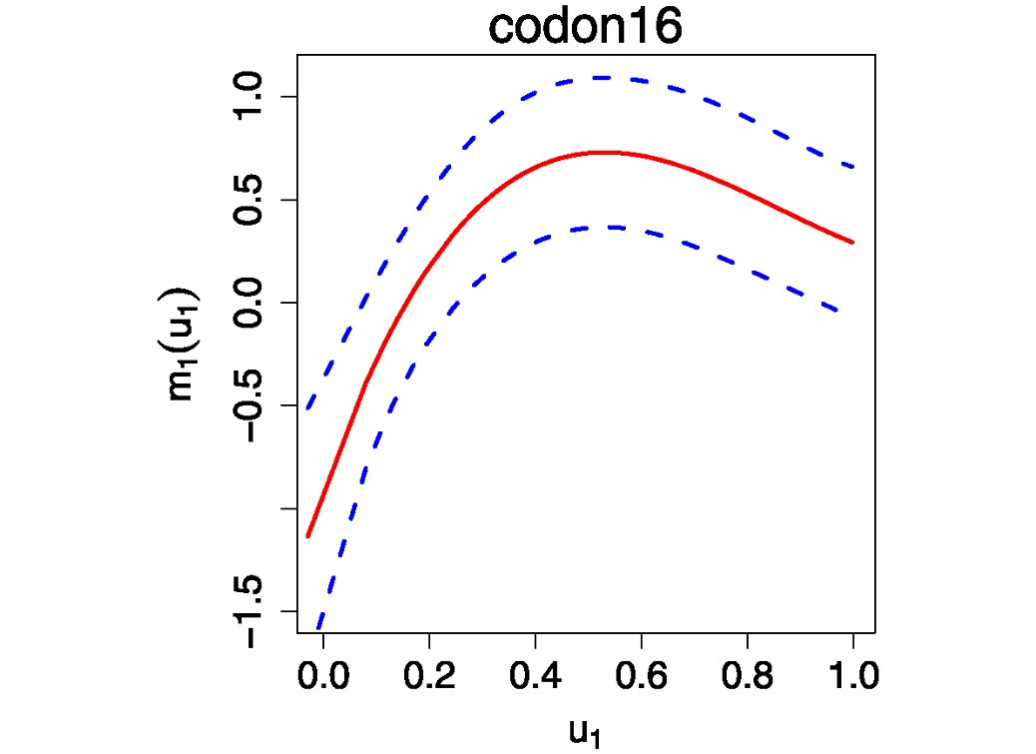
Plot of the estimated nonparametric function *m*_1_(*u*_1_) for SNPs at codons 16, 27, 49, 389, and 492, represented by the solid curve. The 95% confidence bands are shown as dashed lines.

In Table 3, the 5 SNPs have p-values ($p_{m_{1}}$) smaller than the significance level 0.05, which means the functions capturing the G × E interactions are nonlinear for all these 5 SNPs. The objective function *Q*_*N*_ shows that gFVICM provides a better fit to the data than gAVCM does. This demonstrates the advantage of the integrative analysis. Furthermore, the testing results for gAVCM indicate that the coefficients for interactions are not significant. The results imply that these SNP effects are potentially influenced by a mixture of environmental factors, rather than separately. Fig 7 exhibits the fitted nonlinear curves along with the 95% confidence bands, indicating G × E interactions for each SNP.

**Table 3:** List of SNPs showing MAF, alleles, p-values under different hypotheses, and *Q*_*N*_.

SNP ID	MAF	Alleles	p-value					*Q*_*N*_	
			$p_{m_{1}}$	$p_{\beta_{11}}$	$p_{\beta_{12}}$	$p_{\beta_{13}}$	*p*_*gAVCM*_	gFVICM	gAVCM
codon16	0.395	A/G	0.0033	<1.0E-04	<1.0E-04	0.143	0.2541	8.584	16.3250
codon27	0.375	G/C	<1.0E-04	0.367	<1.0E-04	0.0006	0.5712	4.588	7.0152
codon49	0.157	G/A	0.0144	<1.0E-04	<1.0E-04	0.668	0.2013	7.333	12.6841
codon389	0.377	G/C	0.0009	<1.0E-04	<1.0E-04	0.110	0.7592	5.125	11.5328
codon492	0.329	T/C	<1.0E-04	0.556	<1.0E-04	0.0007	0.483	6.505	7.323

Table 4 displays the estimated odds for different genotypes at different dosage levels. Since dosage level (*X*_1_) does not show significance for SNPs condon27 and condon492, we did not show the estimated odds at different dosage levels for these two SNPs in the table. The changes in the values of odds demonstrate the interaction between SNP and environmental mixtures at different dosage levels. For example, we noted that the odds for genotype AA in SNP *codon16* does not change too much as the dosage level increases, which means that the genetic effect of this genotype remains the same when subjects are exposed to different Dobutamine dosage levels. While for the other two genotypes, there is an increase in the value of odds until dosage level four, indicating increased blood pressure as dosage level increases from 0mcg/min to 20mcg/min. We can see the difference of odds at different dosage levels for an individual carrying different SNP genotypes. Using SNP codon389 as an example, individuals with the GG genotype consistently exhibit odds close to 1 across various dosage levels, suggesting the absence of a SNP × dosage interaction affecting the risk of hypotension. Conversely, individuals with the CC or CG genotype show an increased risk of hypotension as the dosage level rises, followed by a decrease after reaching dosage level 4. This indicates varying genetic responses to different dosage levels and, consequently, a SNP × dosage interaction. This will help scientists to get better understanding of the gene function and how different genotypes respond to the combined effect of the three variables to affect the risk of hypertension.

**Table 4:** Estimated odds for different genotypes at each dosage level.

SNP ID	Genotyoe	Dobutamine dosage level(mcg/min)					
		0	5	10	20	30	40
codon16	AA	0.52	0.51	0.50	0.49	0.48	0.47
	GG	0.60	0.80	0.95	1.00	0.84	0.65
	GA	0.68	1.25	1.79	2.07	1.47	0.91
codon49	GG	1.79	1.77	1.75	1.73	1.73	1.74
	AA	1.00	1.28	1.48	1.54	1.34	1.17
	GA	0.56	0.93	1.25	1.36	1.04	0.79
codon389	GG	1.10	1.08	1.06	1.02	1.00	0.99
	CC	0.67	0.89	1.05	1.12	0.98	0.82
	CG	0.41	0.73	1.04	1.23	0.95	0.68

## 6 Discussion

In this paper, we introduced a generalized varying index coefficient modeling approach designed to evaluate the combined interaction effects of multiple environmental factors with a genetic factor. This model was inspired by empirical evidence and developed under a longitudinal design with a binary disease response. We developed a profile estimation procedure to estimate the index coefficients and nonparametric interaction functions iteratively. The estimation was conducted under the QIF framework. To estimate the nonparametric functions, we first approximated the function using truncated power spline basis, then estimated the spline coefficients under the QIF framework. Furthermore, we proposed a hypothesis test to assess the linearity of the nonparametric interaction function. Simulation study has been conducted to illustrate the estimation and testing procedures to evaluate the finite sample performance. The results indicate reasonable estimation performance of the method under different sample sizes and measurement times.

Our method was proposed to evaluate the joint interaction effect between genetic variants and multiple environmental variables as a whole. Compared to the generalized additive varying coefficient model (gAVCM), which models the G × E effect for each single environmental factor separately, our model presents two advantages: 1) it is biologically more attractive if there are synergistic effects between multiple exposures; and 2) it can potentially increase the testing power for detecting interactions since it can reduce multiple testing burden by treating multiple exposures as a single index variable. Although our method was motivated by a genetic association study, the developed model and inference procedures can be applied to other disciplines with the purpose to model the synergistic effect of multiple variables as a whole.

We applied our method to a real data set from a pain sensitivity study. Testing results indicate that all of the five SNPs are nonlinear moderated, by the synergistic effect of the three variables with dosage as a “time”-varying variable, to affect the risk of hypertension. These five SNPs were genotyped from a candidate gene which has been shown to be related to blood pressure changes [31]. Although the purpose of the data was not generated to evaluate the genetic effect on hypertension, we applied the method to this data set to demonstrate the utility of the method. The estimated odds of different genotypes for a particular SNP at different dosage levels does give insights into the effect of the SNPs nonlinearly modulated by different levels of Dobutamine dosage. Of particular interest is SNP condon49 in which individuals carrying genotype GG show a constant higher risk of developing hypertension regardless of the dosage change, indicating no SNP × dosage interaction. For the same SNP, individuals carrying genotype GA show a different pattern of developing hypertension as the dosage level increases. Such a dynamic change of genetic effect over different dosage levels cannot be revealed by a cross-sectional study, indicating the relative merit of a longitudinal design.

Other methods such as the random effects models [32] and the transition models [33] are also choices for longitudinal data analysis. The random effect models account for both fixed effects (which are common to all individuals) and random effects (which capture individual-specific variation) to handle the correlation between repeated measures within the same subject. The transition models focus on modeling the relationship between successive observations over time. This method is well-suited for data where the primary interest is in understanding how the outcome at one time point depends on previous outcomes, such as in Markov models or autoregressive models. We used QIF instead of random effects or transition models in this work due to its robustness to correlation structure misspecification and its ability to provide more efficient parameter estimates.

We also recognize that the present real data analysis is constrained by the examination of a limited number of SNPs. Our ability to access a large-scale longitudinal GWAS dataset is restricted, which affects the scope of our analysis. When working with a substantial number of SNPs, it is crucial to consider a rigorous approach for controlling the false discovery rate (FDR) in multiple testing correction. Nevertheless, it is important to emphasize that our method offers a novel and valuable strategy for conducting synergistic G × E studies within a longitudinal design. It contributes to the expanding toolkit available for G × E analysis, demonstrating its potential for uncovering meaningful insights in genetic research. In addition, missing values are often reported in longitudinal studies. An extension of the work is possible with missing values under the proposed QIF framework, which will be evaluated in our future studies.

## Supporting information

S1 Data Simulation and real data codes to replicate the results in the paper. (ZIP)

S1 File Theorems and proofs. (PDF)
