# Supplementary material for: Generalized functional varying-index coefficient model for dynamic synergistic gene-environment interactions with binary longitudinal traits
Source: PLoS One. 2025 Jan 27;20(1):e0318103. doi: 10.1371/journal.pone.0318103 (PMC11771918; doi:10.1371/journal.pone.0318103)
Supplement: S1 File — (PDF) [file pone.0318103.s002.pdf]

Supplemental file for “Generalized functional varying-index coefficient model for dynamic synergistic gene-environment interactions with binary longitudinal traits”

Jingyi Zhang[1,2*], Honglang Wang[3*] and Yuehua Cui[1†]

[1]*Department of Statistics and Probability, Michigan State University, East Lansing, MI 48824*

[2]*Amazon Lab126, Sunnyvale, CA 94089*

[3]*Department of Mathematical Sciences, Indiana University-Purdue University Indianapolis, Indianapolis, IN 46202*

This supplemental file contains the theories and proofs presented in the main text.

Theorems and Proofs

Theoretical results

To establish the asymptotic properties for the estimators of the index loading parameters and the penalized spline regression coefficients, we assume $\boldsymbol{\theta}_0$ is the parameter satisfying $E_{\boldsymbol{\theta}_0}(g_i) = 0$. Theorem S1 provides the consistency of the resulting estimators. We show the $\sqrt{N}$-consistency and asymptotic normality of the estimators in Theorem S2.

First, to handle the constraints $\|\boldsymbol{\beta}_0\|$=$\|\boldsymbol{\beta}_1\|$=1, and $\beta_{01} > 0$, $\beta_{11} > 0$, we set $\beta_{l1} = \sqrt{1 - \|\boldsymbol{\beta}_{l,-1}\|_2^2}$ with $\boldsymbol{\beta}_{l,-1} = (\beta_{l2}, ..., \beta_{lp})^T$ for l=1, 2. Then the parameter space of $\boldsymbol{\beta}_l$, l=1,2,

*The first two authors contributed equally to this work

†To whom correspondence should be addressed: cuiy@msu.edu

becomes

$$[\{(\sqrt{1-\|\boldsymbol{\beta}_{l,-1}\|_2^2}, \beta_{l2}, ..., \beta_{lp})^T\} : \|\boldsymbol{\beta}_{l,-1}\|_2^2 < 1].$$

Let

$$\mathbf{J}_l = \frac{\partial \boldsymbol{\beta}_l}{\partial \boldsymbol{\beta}_{l,-1}^T} = \begin{pmatrix} -\boldsymbol{\beta}_{l,-1}^T / \sqrt{1-\|\boldsymbol{\beta}_{l,-1}\|_2^2} \\ \mathbf{I}_{p-1} \end{pmatrix}$$

be the Jacobian matrix of dimension $p \times (p-1)$. Denote $\boldsymbol{\beta}_{-1} = (\boldsymbol{\beta}_{0,-1}^T, \boldsymbol{\beta}_{1,-1}^T)^T$, and $\boldsymbol{\theta}^* = (\boldsymbol{\beta}_{-1}, \boldsymbol{\gamma})^T$. From $\boldsymbol{\theta}$ to $\boldsymbol{\theta}^*$, we have Jacobian matrix $\mathbf{J} = \text{diag}(\mathbf{J}_0, \mathbf{J}_1, \mathbf{I}_{q+K+1}, \mathbf{I}_{q+K+1})$.

Theorem S1. *Suppose the assumptions (A1)-(A6) in the Appendix are satisfied and the smoothing parameter $\lambda_N = o(1)$, then the estimator $\widehat{\boldsymbol{\theta}}$, which is obtained by minimizing the penalized quadratic inference function in (4) in the main text, exists and converges to $\boldsymbol{\theta}_0$ in probability.*

Theorem S2. *Suppose the assumptions (A1)-(A6) in the Appendix are satisfied, and the smoothing parameter $\lambda_N = o(N^{-1/2})$, then the estimator $\widehat{\boldsymbol{\theta}}$ obtained by minimizing the penalized quadratic inference function in (4) in the main text is asymptotically normally distributed, i.e.,*

$$\sqrt{N}(\widehat{\boldsymbol{\theta}} - \boldsymbol{\theta}_0) \xrightarrow{d} N(\boldsymbol{0}, \boldsymbol{J}(\boldsymbol{G}_0^T \boldsymbol{C}_0^{-1} \boldsymbol{G}_0)^{-1} \boldsymbol{J}^T),$$

where the detailed calculation of $\boldsymbol{G}_0$ and $\boldsymbol{C}_0$ is given in the Appendix.

The asymptotic normality provides the basis for testing the effect of the loading parameters in $\boldsymbol{\beta}_1$. The proof of the theorems follows our previous work by Wang et al. (2022). To establish the asymptotic properties for the estimator of $\boldsymbol{\theta}$, we need the following regularity conditions.

(A1) $\{n_i\}$ is a bounded sequence of integers.

(A2) The parameter space Ω is compact and $\boldsymbol{\theta}_0^*$ is an interior point of Ω.

(A3) The parameter $\boldsymbol{\theta}^*$ is identifiable, that is, there is a unique $\boldsymbol{\theta}_0^* \in \Omega$ such that the mean zero model assumption $E[g(\boldsymbol{\theta}_0^*)] = 0$ is satisfied.

(A4) $E[g(\boldsymbol{\theta})]$ is continuous in $\boldsymbol{\theta}$.

(A5) $\bar{C}_N(\widehat{\boldsymbol{\theta}}^*) = \frac{1}{N}\sum_{i=1}^N g_i(\widehat{\boldsymbol{\theta}}^*) g_i(\widehat{\boldsymbol{\theta}}^*)^T$ converges almost surely to $\mathbf{C}_0$, which is a constant and invertible matrix.

(A6) The first derivative of $\bar{g}_N$ exists and is continuous. $\frac{\partial \bar{g}_N}{\partial \boldsymbol{\theta}^*}(\widehat{\boldsymbol{\theta}}^*)$ converges in probability to $\mathbf{G}_0$ if $\widehat{\boldsymbol{\theta}}^*$ converges in probability to $\boldsymbol{\theta}_0^*$.

Proof of Theorem S1: If we can prove that $\widehat{\boldsymbol{\theta}}^*$ exist and converges to $\boldsymbol{\theta}_0^*$ almost surely, then we can prove the consistency of $\boldsymbol{\theta}$ directly. $\widehat{\boldsymbol{\theta}}^* = \arg\min(N^{-1}Q_N(\boldsymbol{\theta}^*) + \lambda \boldsymbol{\theta}^{*T}\mathbf{D}\boldsymbol{\theta}^*)$ exists because (4) (in the main text) has zero as a lower bound and the global minimum exists. To prove the consistency, first, the estimator $\widehat{\boldsymbol{\theta}}^*$ is obtained by minimizing $N^{-1}Q_N(\boldsymbol{\theta}^*) + \lambda \boldsymbol{\theta}^{*T}\mathbf{D}\boldsymbol{\theta}^*$, then we have

$$\frac{1}{N}Q_N(\widehat{\boldsymbol{\theta}}^*) + \lambda_N \widehat{\boldsymbol{\theta}}^{*T} D \widehat{\boldsymbol{\theta}}^* \le \frac{1}{N}Q_N(\boldsymbol{\theta}_0^*) + \lambda_N \boldsymbol{\theta}_0^{*T} D \boldsymbol{\theta}_0^*. \tag{S.1}$$

Since

$$\frac{1}{N}Q_N(\boldsymbol{\theta}_0^*) = \bar{g}_N^T(\boldsymbol{\theta}_0^*)\bar{C}_N^{-1}(\boldsymbol{\theta}_0^*)\bar{g}_N(\boldsymbol{\theta}_0^*) = o(1)$$

by the strong law of large number and (A5), and $\lambda_N = o(1)$,

$$\frac{1}{N}Q_N(\boldsymbol{\theta}_0^*) + \lambda_N \boldsymbol{\theta}_0^{*T} D \boldsymbol{\theta}_0^* \xrightarrow{a.s.} 0.$$

Thus, we can obtain from (S.1) that

$$\frac{1}{N}Q_N(\widehat{\boldsymbol{\theta}}^*) = \bar{g}_N^T(\widehat{\boldsymbol{\theta}}^*)\bar{C}_N^{-1}(\widehat{\boldsymbol{\theta}}^*)\bar{g}_N(\widehat{\boldsymbol{\theta}}^*) \xrightarrow{a.s.} 0. \tag{S.2}$$

Since the parameter space Ω is compact, by Glivenko-Cantelli theorem,

$$\sup_{\theta^* \in \Omega} \left| \bar{g}_N(\boldsymbol{\theta}^*) - E[g(\boldsymbol{\theta}^*)] \right| \xrightarrow{a.s.} 0.$$

Hence, by (A5) and the continuous mapping theorem,

$$\left| \bar{g}_N^T(\widehat{\boldsymbol{\theta}}^*)\bar{C}_N^{-1}(\widehat{\boldsymbol{\theta}}^*)\bar{g}_N(\widehat{\boldsymbol{\theta}}^*) - E[g(\widehat{\boldsymbol{\theta}}^*)]^T \mathbf{C}_0^{-1} E[g(\widehat{\boldsymbol{\theta}}^*)] \right| \xrightarrow{a.s.} 0.$$

Combined with (S.2), we get

$$E[g(\widehat{\boldsymbol{\theta}}^*)]^T \mathbf{C}_0^{-1} E[g(\widehat{\boldsymbol{\theta}}^*)] \xrightarrow{a.s.} 0. \tag{S.3}$$

Next, we will show that it is impossible that $\widehat{\boldsymbol{\theta}}^*$ remains outside of U, where U is any neighborhood of the true parameter $\boldsymbol{\theta}_0^*$. Suppose there exists a neighborhood U such that $\widehat{\boldsymbol{\theta}}^* \in U^c$. Since $E[g(\boldsymbol{\theta}^*)]^T \mathbf{C}_0^{-1} E[g(\boldsymbol{\theta}^*)]$ is a continuous function and U^c is compact, there

exists a point $\widetilde{\boldsymbol{\theta}}^* \in U^c$ such that $E[g(\widetilde{\boldsymbol{\theta}}^*)]^T \mathbf{C}_0^{-1} E[g(\widetilde{\boldsymbol{\theta}}^*)]$ achieves its minimum in U^c. By the identifiability of $\boldsymbol{\theta}^*$ in (A3), there is a unique $\boldsymbol{\theta}_0^* \in \Omega$ satisfying $E[g(\boldsymbol{\theta}_0)] = 0$, so we have

$$E[g(\boldsymbol{\theta}^*)]^T \mathbf{C}_0^{-1} E[g(\boldsymbol{\theta}^*)] > 0,$$

which contradicts $(S.3)$. Then we can prove that $\widehat{\boldsymbol{\theta}}^*$ converges almost surely to $\boldsymbol{\theta}^*$. Thus, $\widehat{\boldsymbol{\theta}}$ is a consistent estimator of $\boldsymbol{\theta}$.

Proof of Theorem S2: The estimate of $\boldsymbol{\theta}$ satisfies

$$0 = \frac{1}{N}\frac{\partial Q_N}{\partial \boldsymbol{\theta}^*}(\widehat{\boldsymbol{\theta}}^*) + 2\lambda_N D\widehat{\boldsymbol{\theta}}^*.$$

By Taylor expansion, we obtain

$$0 = \frac{1}{N}\frac{\partial Q_N}{\partial \boldsymbol{\theta}}^*(\boldsymbol{\theta}_0^*) + 2\lambda_N D\boldsymbol{\theta}_0^* + \Big(\frac{1}{N}\frac{\partial^2 Q_N}{\partial \boldsymbol{\theta}^{*2}}(\widetilde{\boldsymbol{\theta}}^*) + 2\lambda_N D\Big)(\widehat{\boldsymbol{\theta}}^* - \boldsymbol{\theta}_0^*),$$

where $\widetilde{\boldsymbol{\theta}}^*$ is some value between $\widehat{\boldsymbol{\theta}}^*$ and $\boldsymbol{\theta}_0^*$. Thus, we can have

$$\widehat{\boldsymbol{\theta}}^* - \boldsymbol{\theta}_0^* = -\Big(\frac{1}{N}\frac{\partial^2 Q_N}{\partial \boldsymbol{\theta}^{*2}}(\widetilde{\boldsymbol{\theta}}^*) + 2\lambda_N D\Big)^{-1}\Big(\frac{1}{N}\frac{\partial Q_N}{\partial \boldsymbol{\theta}^*}(\boldsymbol{\theta}_0^*) + 2\lambda_N D\boldsymbol{\theta}_0^*\Big). \tag{S.4}$$

Since $\widehat{\boldsymbol{\theta}}^*$ converges to $\boldsymbol{\theta}_0^*$ in probability and $\widetilde{\boldsymbol{\theta}}^*$ is between $\widehat{\boldsymbol{\theta}}^*$ and $\boldsymbol{\theta}_0^*$, by (A5) and (A6) we can get

$$\begin{aligned}\frac{1}{N}\frac{\partial^2 Q_N}{\partial \boldsymbol{\theta}^{*2}}(\widetilde{\boldsymbol{\theta}}^*) \quad &= \quad 2\frac{\partial \bar{g}_N}{\partial \boldsymbol{\theta}^*}^T(\widetilde{\boldsymbol{\theta}}^*)\bar{C}_N^{-1}(\widetilde{\boldsymbol{\theta}}^*)\frac{\partial \bar{g}_N}{\partial \boldsymbol{\theta}^*}(\widetilde{\boldsymbol{\theta}}^*) + o_p(1) \\ &\xrightarrow{\mathcal{P}} \quad 2\mathbf{G}_0^T\mathbf{C}_0^{-1}\mathbf{G}_0\end{aligned}$$

When $\lambda_N = o(N^{-1/2})$,

$$\Big(\frac{1}{N}\frac{\partial^2 Q_N}{\partial \boldsymbol{\theta}^{*2}}(\widetilde{\boldsymbol{\theta}}^*) + 2\lambda_N D\Big)^{-1} = \frac{1}{2}(\mathbf{G}_0^T\mathbf{C}_0^{-1}\mathbf{G}_0)^{-1} + o_p(N^{-1/2}).$$

Similarly, since

$$\frac{1}{N}\frac{\partial Q_N}{\partial \boldsymbol{\theta}^*}(\boldsymbol{\theta}_0^*) = \frac{\partial \bar{g}_N}{\partial \boldsymbol{\theta}^*}^T(\boldsymbol{\theta}_0^*)\bar{C}_N^{-1}(\boldsymbol{\theta}_0^*)\bar{g}_N(\boldsymbol{\theta}_0^*)$$

and $\lambda_N = o(N^{-1/2})$, we have

$$\frac{1}{N}\frac{\partial Q_N}{\partial \boldsymbol{\theta}^*}(\boldsymbol{\theta}_0^*) + 2\lambda_N D\boldsymbol{\theta}_0^* = G_0^T C_0^{-1}\bar{g}_N(\boldsymbol{\theta}_0^*) + o(N^{-1/2}).$$

Therefore, (S.4) can be written as

$$\sqrt{N}(\widehat{\boldsymbol{\theta}}^* - \boldsymbol{\theta}_0^*) = -\sqrt{N}(\mathbf{G}_0^T\mathbf{C}_0^{-1}\mathbf{G}_0)^{-1}\mathbf{G}_0^T\mathbf{C}_0^{-1}\bar{g}_N(\boldsymbol{\theta}_0^*) + o_p(1). \tag{S.5}$$

By the Central Limit Theorem,

$$\sqrt{N}\bar{g}_N(\boldsymbol{\theta}_0^*) \xrightarrow{\mathcal{D}} N(\mathbf{0}, \mathbf{C}_0). \tag{S.6}$$

Using (S.5) and (S.6), we obtain

$$\sqrt{N}(\widehat{\boldsymbol{\theta}}^* - \boldsymbol{\theta}_0^*) \xrightarrow{\mathcal{D}} N(\mathbf{0}, (\mathbf{G}_0^T \mathbf{C}_0^{-1} \mathbf{G}_0)^{-1}),$$

and directly,

$$\sqrt{N}(\widehat{\boldsymbol{\theta}} - \boldsymbol{\theta}_0) \xrightarrow{\mathcal{D}} N(\mathbf{0}, \mathbf{J}(\mathbf{G}_0^T \mathbf{C}_0^{-1} \mathbf{G}_0)^{-1} \mathbf{J}^T).$$

References

[1] Wang, H., J. Zhang, K.L. Klump, S. Alexandra Burt, Y. Cui. (2022) Multivariate partial linear varying coefficients model for gene-environment interactions with multiple longitudinal traits. *Stat. Med.* **41**, 3643-3660.
